# Supplementary material for: The defensome of prokaryotes in aquifers
Source: Nat Commun. 2025 Jul 14;16:6482. doi: 10.1038/s41467-025-61467-w (PMC12259885; doi:10.1038/s41467-025-61467-w)
Supplement: Supplementary file 1 — Supplementary Information [file 41467_2025_61467_MOESM1_ESM.pdf]

Supplementary Information

The defensome of prokaryotes in aquifers

Pengwei Li[1,2], Zongzhi Wu[1,3], Tang Liu[4], Chunfang Deng[5], Quan Liu[6], Jinren Ni[1,5*]

[1]Environmental Microbiome and Innovative Genomics Laboratory, College of Environmental Sciences and Engineering, Peking University, Beijing 100871, P. R. China;

[2]College of Environmental Sciences and Engineering, Key Laboratory of Water and Sediment Sciences, Ministry of Education, Peking University, Beijing 100871, P. R. China;

[3]State Environmental Protection Key Laboratory of All Materials Fluxes in River Ecosystems, Beijing 100871, P. R. China;

[4]Environmental Microbiome Engineering and Innovative Genomics Laboratory, College of Chemistry and Environmental Engineering, Shenzhen University, Shenzhen 518060, P. R. China;

[5]Eco-environment and Resource Efficiency Research Laboratory, School of Environment and Energy, Peking University Shenzhen Graduate School, Shenzhen 518055, P. R. China;

[6]Peking-Tsinghua Center for Life Sciences, State Key Laboratory of Gene Function and Modulation Research, Peking-Tsinghua-NIBS Graduate Program, School of Life Sciences, Peking University, Beijing 100871, P. R. China.

[*] Corresponding author: Jinren Ni (E-mail: jinrenni@pku.edu.cn)

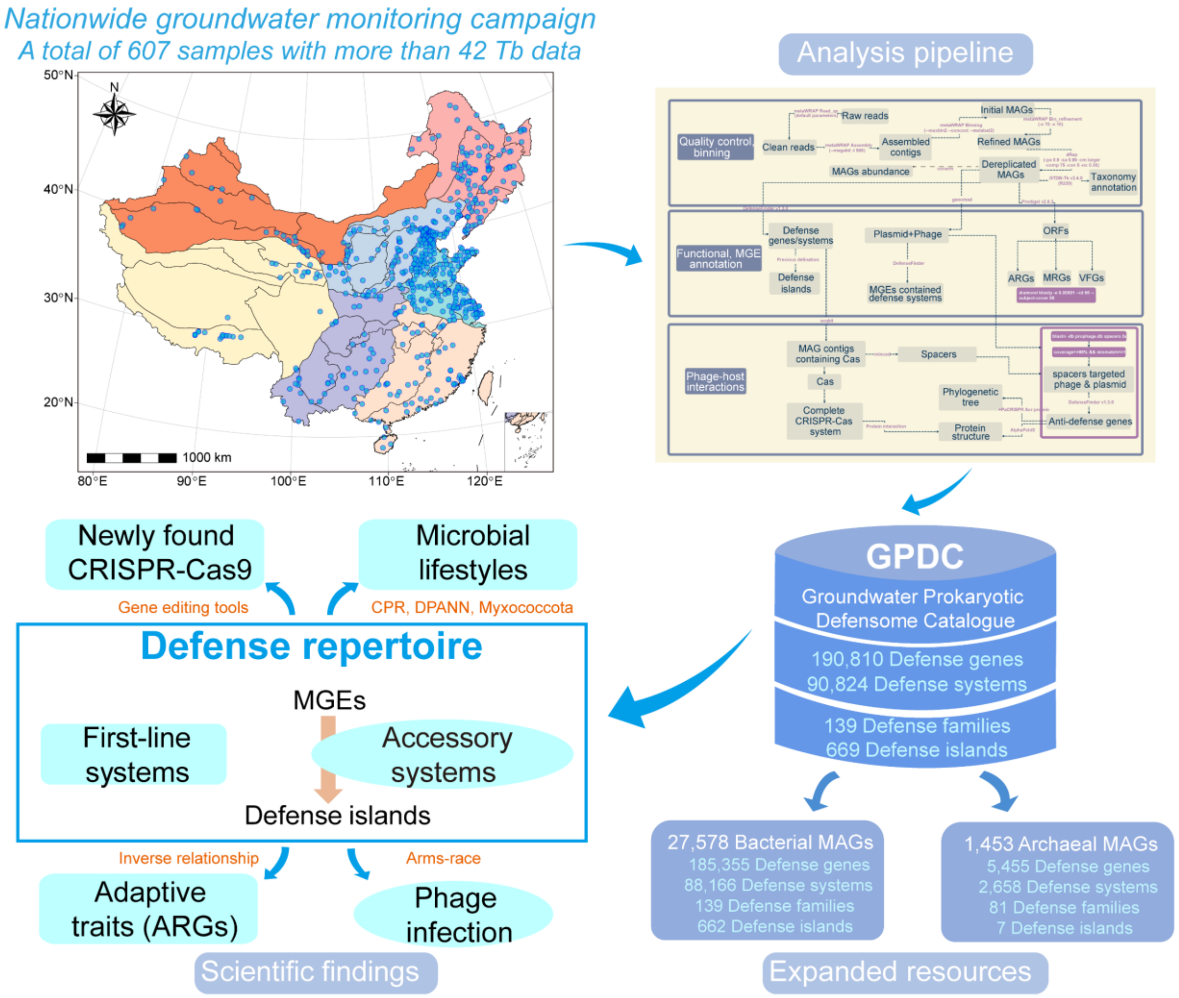

Nationwide groundwater monitoring campaign
A total of 607 samples with more than 42 Tb data
50°N
40°N
30°N
20°N
N
1000 km
80°E
90°E
100°E
110°E
120°E
Analysis pipeline
GPDC
Groundwater Prokaryotic Defensome Catalogue
190,810 Defense genes
90,824 Defense systems
139 Defense families
669 Defense islands
27,578 Bacterial MAGs
185,355 Defense genes
88,166 Defense systems
139 Defense families
662 Defense islands
1,453 Archaeal MAGs
5,455 Defense genes
2,658 Defense systems
81 Defense families
7 Defense islands
Expanded resources
Newly found CRISPR-Cas9
Microbial lifestyles
Gene editing tools
CPR, DPANN, Myxococcota
Defense repertoire
MGEs
First-line systems
Accessory systems
Defense islands
Inverse relationship
Arms-race
Adaptive traits (ARGs)
Phage infection
Scientific findings

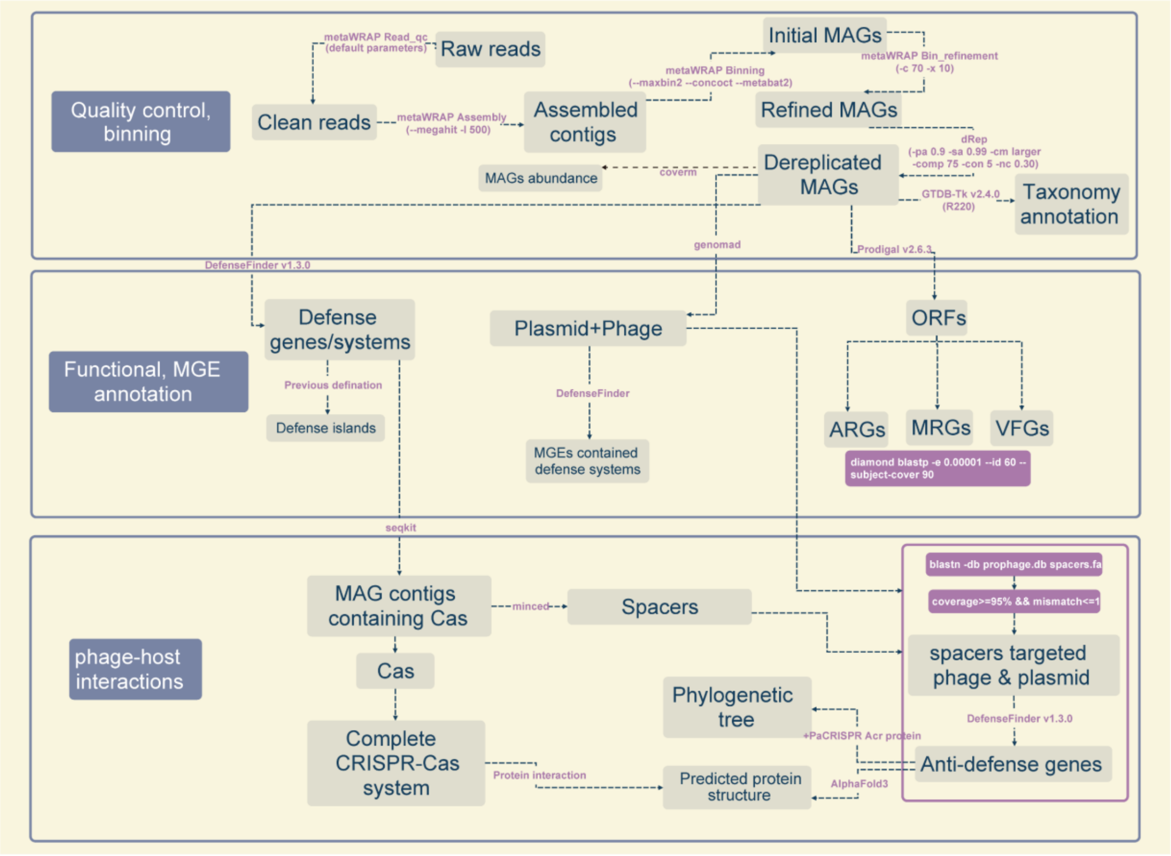

Quality control, binning
Raw reads
metaWRAP Read_qc (default parameters)
Clean reads
metaWRAP Assembly (--megahit -l 500)
Assembled contigs
metaWRAP Binning (--maxbin2 --concoct --metabat2)
Initial MAGs
metaWRAP Bin_refinement (-c 70 -x 10)
Refined MAGs
dRep (-pa 0.9 -sa 0.99 -cm larger -comp 75 -con 5 -nc 0.30)
Dereplicated MAGs
coverm
MAGs abundance
GTDB-Tk v2.4.0 (R220)
Taxonomy annotation
genomad
Prodigal v2.6.3
DefenseFinder v1.3.0
Functional, MGE annotation
Defense genes/systems
Previous defination
Defense islands
Plasmid+Phage
DefenseFinder
MGEs contained defense systems
ORFs
ARGs
MRGs
VFGs
diamond blastp -e 0.00001 --id 60 --subject-cover 90
seqkit
phage-host interactions
MAG contigs containing Cas
minced
Spacers
Cas
Complete CRISPR-Cas system
Protein interaction
Predicted protein structure
AlphaFold3
Phylogenetic tree
+PaCRISPR Acr protein
blastn -db prophage.db spacers.fa
coverage>=95% && mismatch<=1
spacers targeted phage & plasmid
DefenseFinder v1.3.0
Anti-defense genes

Fig. S1. Overview and workflow of bioinformatic analyses. This figure presents the groundwater prokaryotic defensome catalogue (GPDC) and the findings derived from it, along with detailed bioinformatic analysis processes.

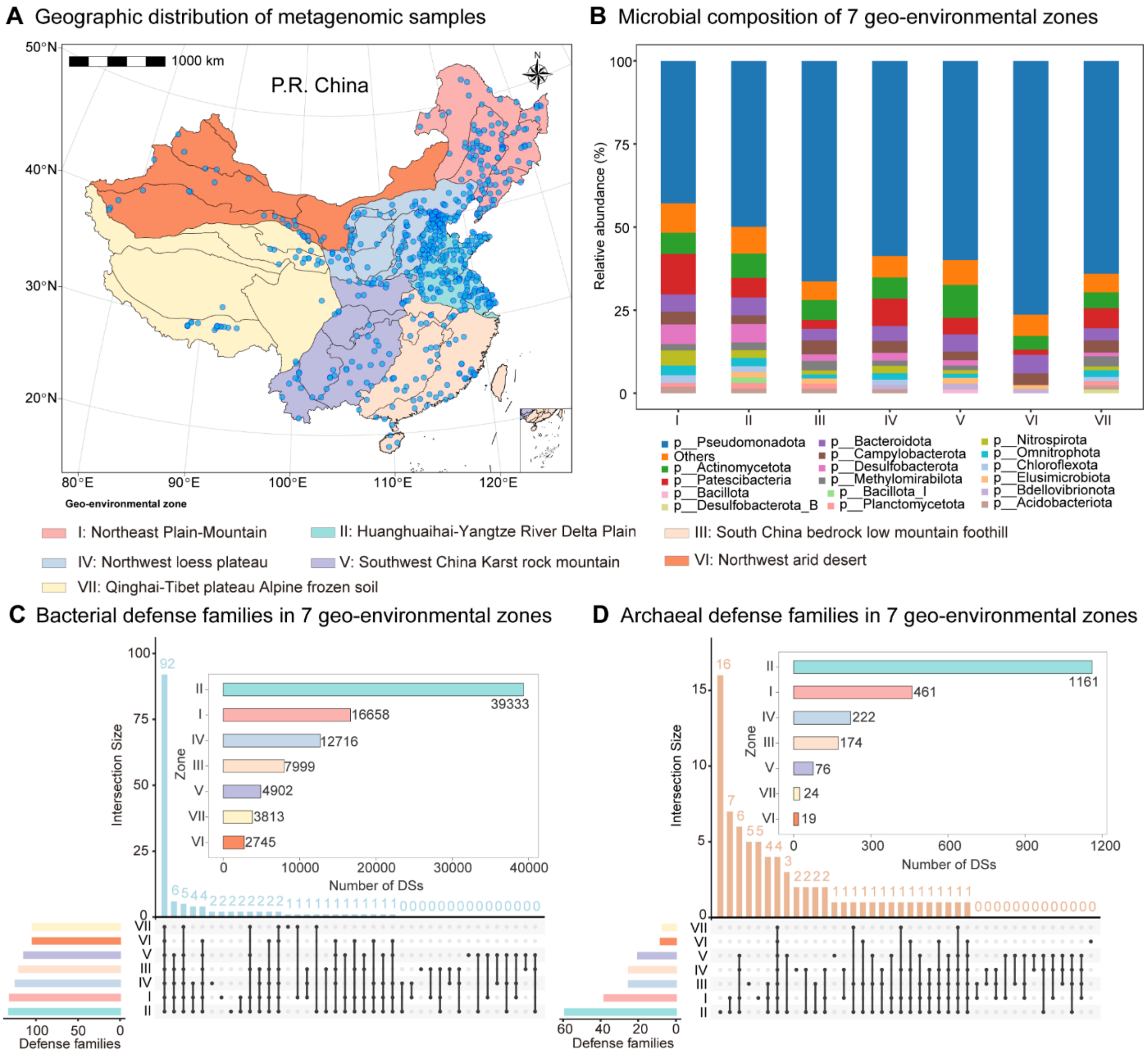

Fig. S2. Geographic distribution of the GPDC. (A) Sample site locations. **(B)** Relative abundance of microbial phyla across seven geographic zones. **(C)** Occurrence of bacterial defense systems in different geo-environmental zones. **(D)** Occurrence of archaeal defense systems in different geo-environmental zones. Source data are provided as a Source Data file.

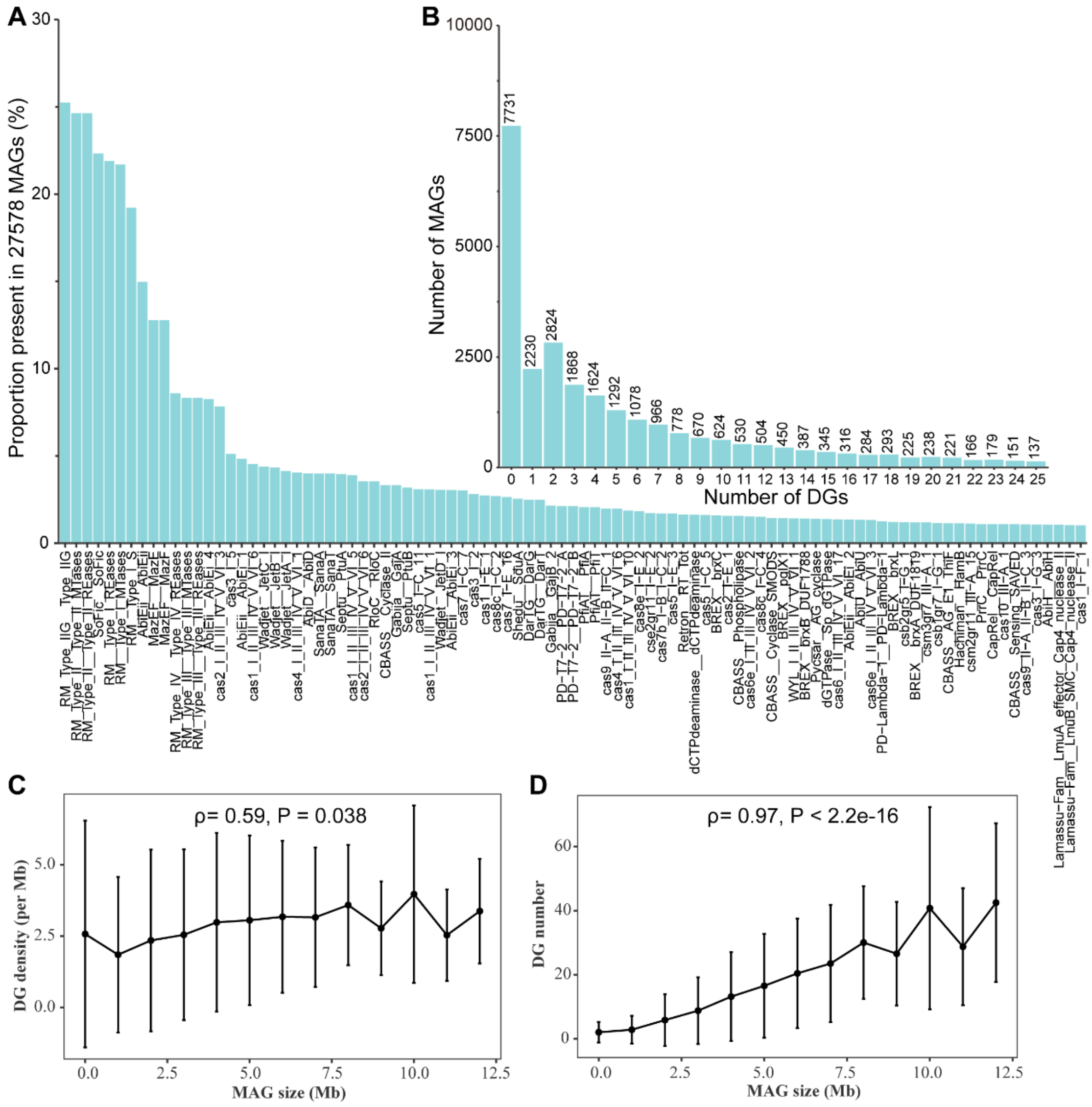

Fig. S3. Distribution of defense genes (DGs) in groundwater bacteria. (A) Prevalence of defense genes in bacterial MAGs, showing only genes present in at least 1% of all MAGs. **(B)** Distribution of defense gene number across all MAGs. **(C) (D)** Variation in defense gene density (per MAG and per Mb) and gene number (per MAG) across 27,578 MAGs. Error bars represent standard deviations of the mean; correlation was assessed by two-sided Spearman's rank test with exact P value provided. Number of MAGs analyzed are same with Fig. 1G. Source data are provided as a Source Data file.

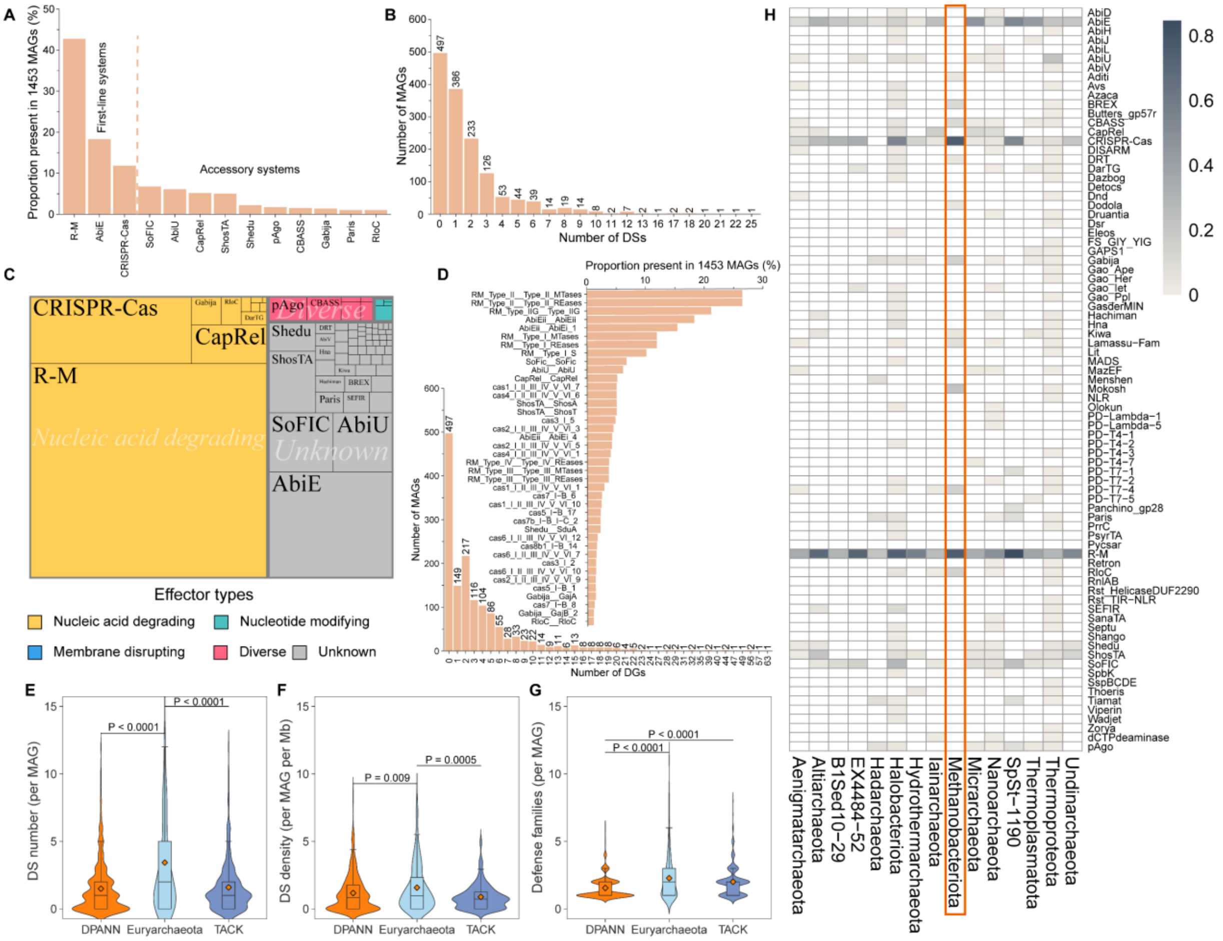

Fig. S4. The defensome of groundwater archaea. (A) Prevalence of defense families in archaeal MAGs, showing only families present in at least 1% of all MAGs. **(B)** Distribution of defense system number per MAG. **(C)** Relative abundance of defense systems in archaeal genomes. Treemap rectangles represent defense systems, with aera proportional to mean abundance and color indicating effector type. **(D)** Prevalence of defense genes in archaeal MAGs, showing only genes present in at least 1% of all MAGs, and distribution of defense gene number across all MAGs. **(E), (F), and (G)** Comparison of defense system number (per MAG), density (per MAG and per Mb), and family diversity among the DPANN, Euryarchaeota, and TACK. Boxplots represent the 25th to 75th percentiles, the inner black line marks the median, whiskers extend to 1.5x the interquartile range, and the orange square dot represents the mean. Significant differences were assessed using the two-sided Wilcox test with Bonferroni-adjusted P values. Analyzed MAG number for DPANN, Euryarchaeota, and TACK is 880, 235, and 338. **(H)** The heatmap shows the presence frequency of each defense system (rows) in each phylum (columns), with colors indicating frequency. Source data are provided as a Source Data file.

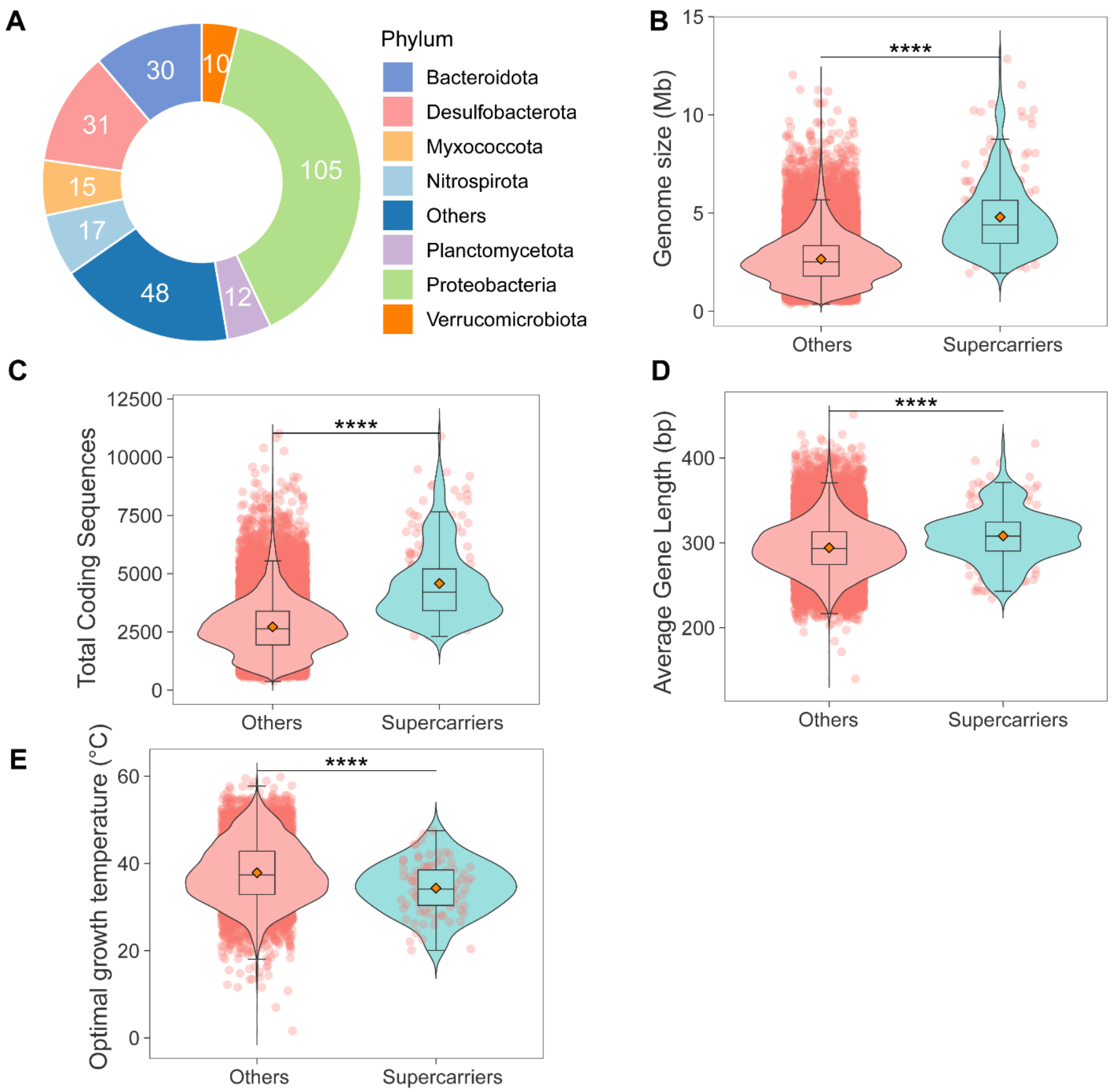

Fig. S5. Bacterial supercarriers of defense systems. (A) Taxonomic composition of the 268 supercarrier genomes. **(B), (C), (D) and (E)** Comparison of genome size (Mb), total coding sequences, average gene length (bp), and optimal growth temperature (°C) between supercarriers and other bacterial genomes. Significant differences were assessed using the two-sided Wilcox test with Bonferroni-adjusted P values. "****" indicates $P < 0.0001$. Analyzed MAG number for Others and Supercarriers is 268 and 27310. Source data are provided as a Source Data file.

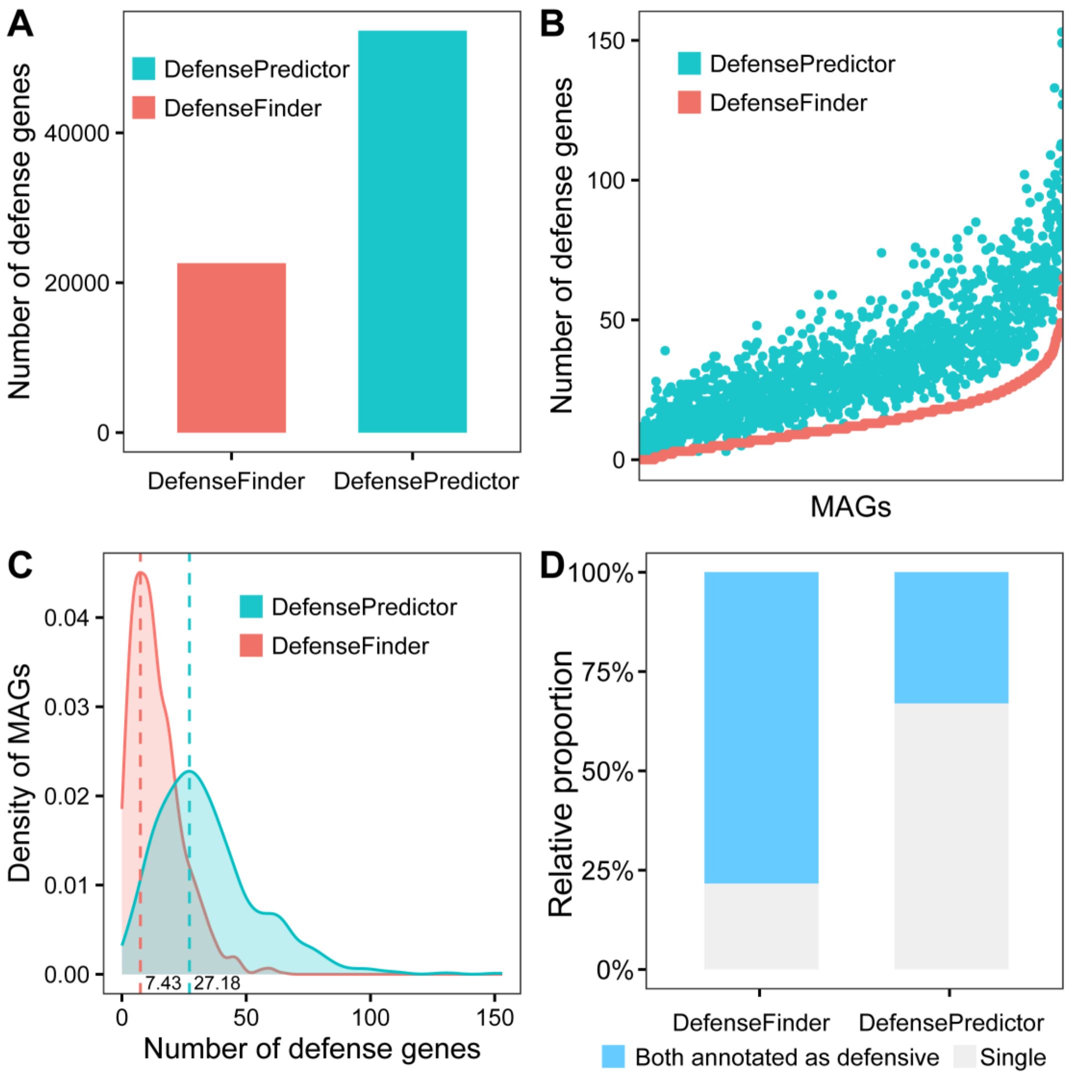

Fig. S6. DefensePredictor results on 1,626 high-quality MAGs. (A) The number of defense genes identified by DefenseFinder and DefensePredictor on 1,626 high-quality MAGs. **(B)** Results derived from the two software on each MAG. The MAGs were sorted according to the DefenseFinder results from the smallest to the largest. **(C)** Density plots showing number proportion of defense genes identified using the two software among MAGs where defense genes were detected. Dashed lines represent the number of defense genes corresponding to the maximum density. **(D)** The proportion of co-identified defense genes (by both software) and those identified with DefenseFinder or DefensePredictor. Source data are provided as a Source Data file.

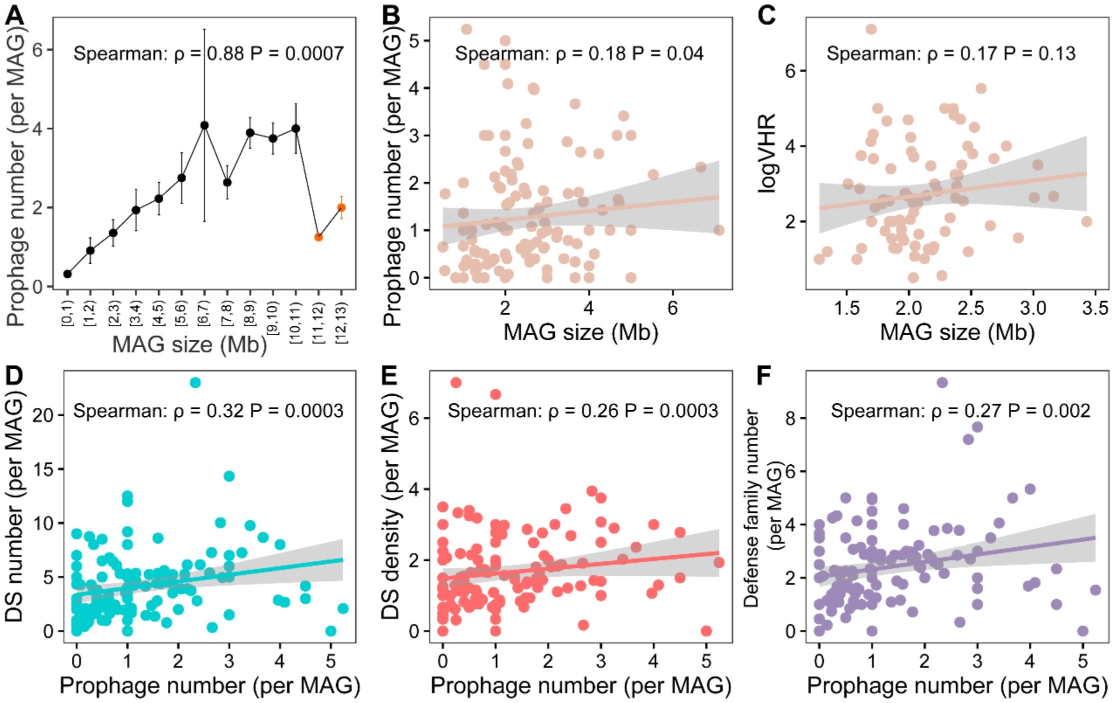

Fig. S7. Effects of genome size on prophage encounter and prophage number on the defensome. (A) Variation in prophage number (per MAG) across all MAGs. Error bars represent standard deviations of the mean; correlation assessed by two-sided Spearman's rank test. To ease visualization, standard errors were divided by 10, and the orange points were excluded from the Spearman test. **(B)** Variation in prophage number (per MAG) across all bacterial phyla. Each dot represents a phylum. **(C)** Relationship between virus-host abundance ratios (VHR) and MAG size. **(D), (E), (F)** Positive correlations between defense system (DS) number, DS density, defense family number, and prophage number. In **B-F**, the central line was added by using "lm" method, with 0.95 confidence intervals marked by gray shading. Correlation was assessed using two-sided Spearman's rank test with exact P value provided. Source data are provided as a Source Data file.

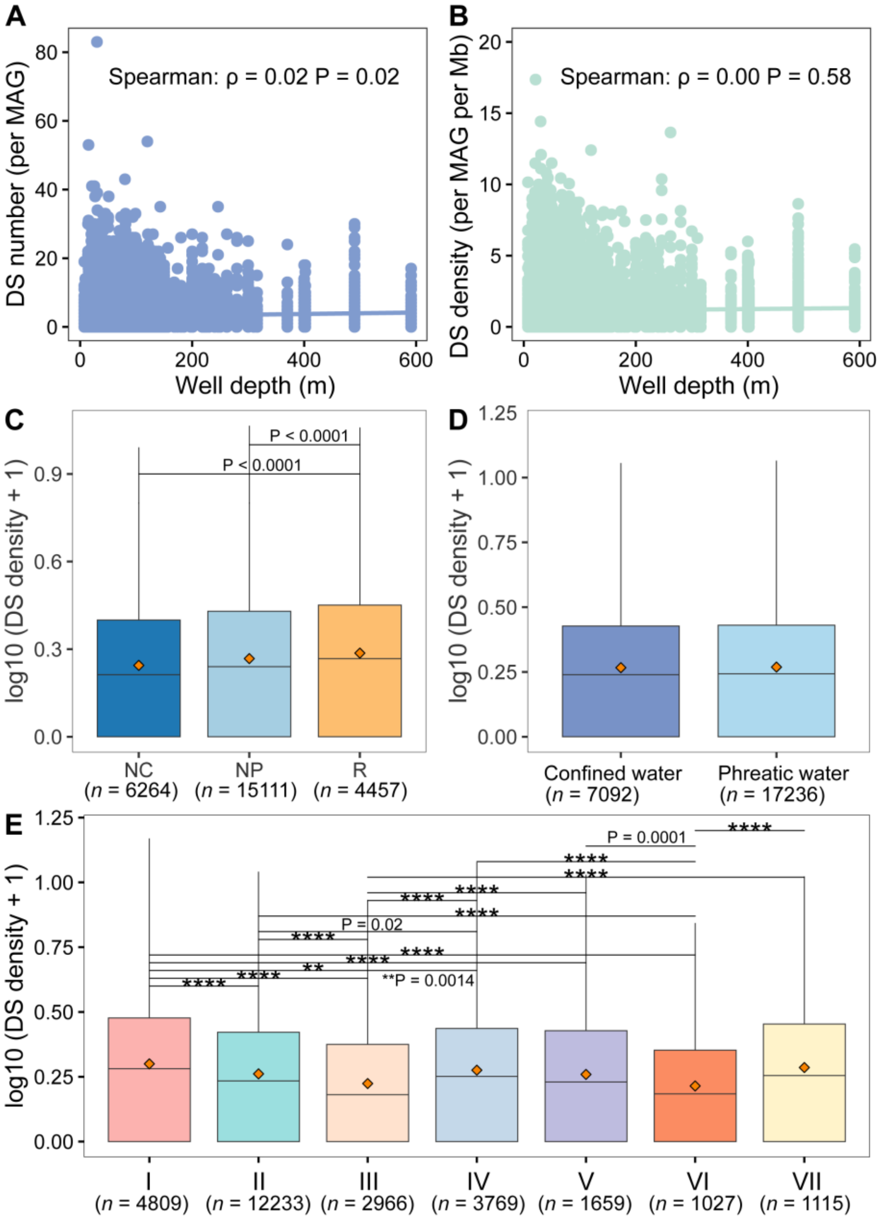

Fig. S8. Effects of sampling depths, well types, and geographical contexts on the microbial defensome. (A) Correlation between defense system (DS) number and burial depth of groundwater. **(B)** Correlation between defense system density (per MAG per Mb) and burial depth of groundwater. In **A-B**, the central line was added by using "lm" method, correlation was assessed using two-sided Spearman's rank test. **(C)** Comparison of defense system density (per MAG per Mb) among newly constructed confined wells (NC), newly constructed phreatic wells (NP), and reconstructed wells (R). **(D)** Comparison of defense system density (per MAG per Mb) in confined water and phreatic water. **(E)** Comparison of defense system density (per MAG per Mb) across seven geographic zones. Detailed zone annotations are provided in Fig. S2A. Number of MAGs analyzed are shown as n values. Boxplots in **C-E** represent the 25th to 75th percentiles, the inner black line marks the median, whiskers extend to 1.5x the interquartile range, and the orange square dot represents the mean. Significant differences were assessed using the two-sided Wilcox test with Bonferroni-adjusted P values. "****" indicates P < 0.0001. Source data are provided as a Source Data file.

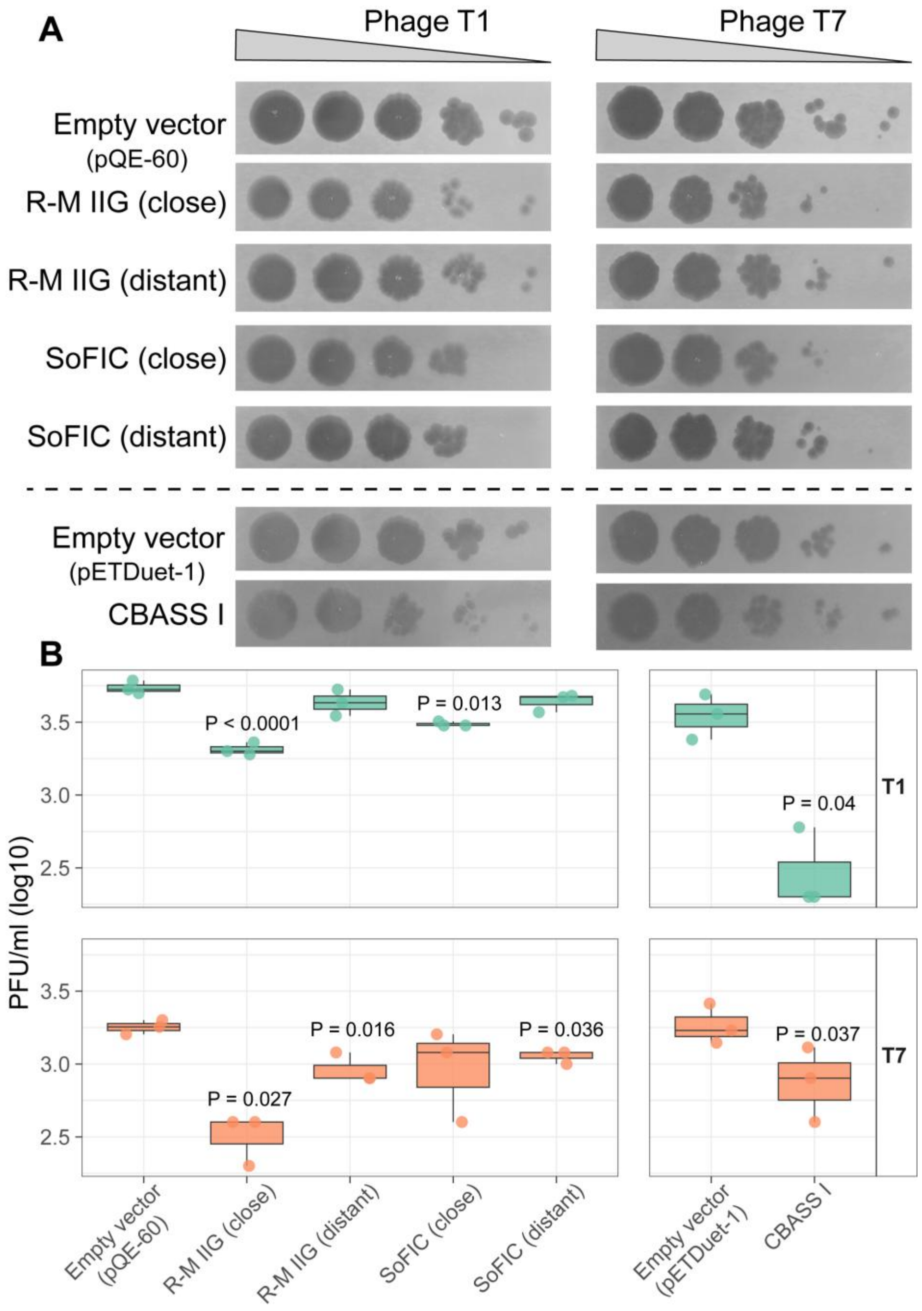

Fig. S9. Plaque assays showing phage infection of *Escherichia coli* B (BL21) transformed with plasmids carrying representative defense systems or empty vector (control). Ten-fold serial dilutions of phages (T1 and T7) were spotted onto bacterial lawns. **(A)** Plaque assay image after incubation at 25°C overnight. **(B)** Viral plaque forming unit (PFU) measured by plaque assay at 7 h post-infection. n = 3 biological replicates. Statistic differences between empty vector and vectors carrying defense systems were assessed using paired two-sided t test. Boxplots represent the 25th to 75th percentiles, the inner black line marks the median, whiskers extend to 1.5x the interquartile range. Source data are provided as a Source Data file.

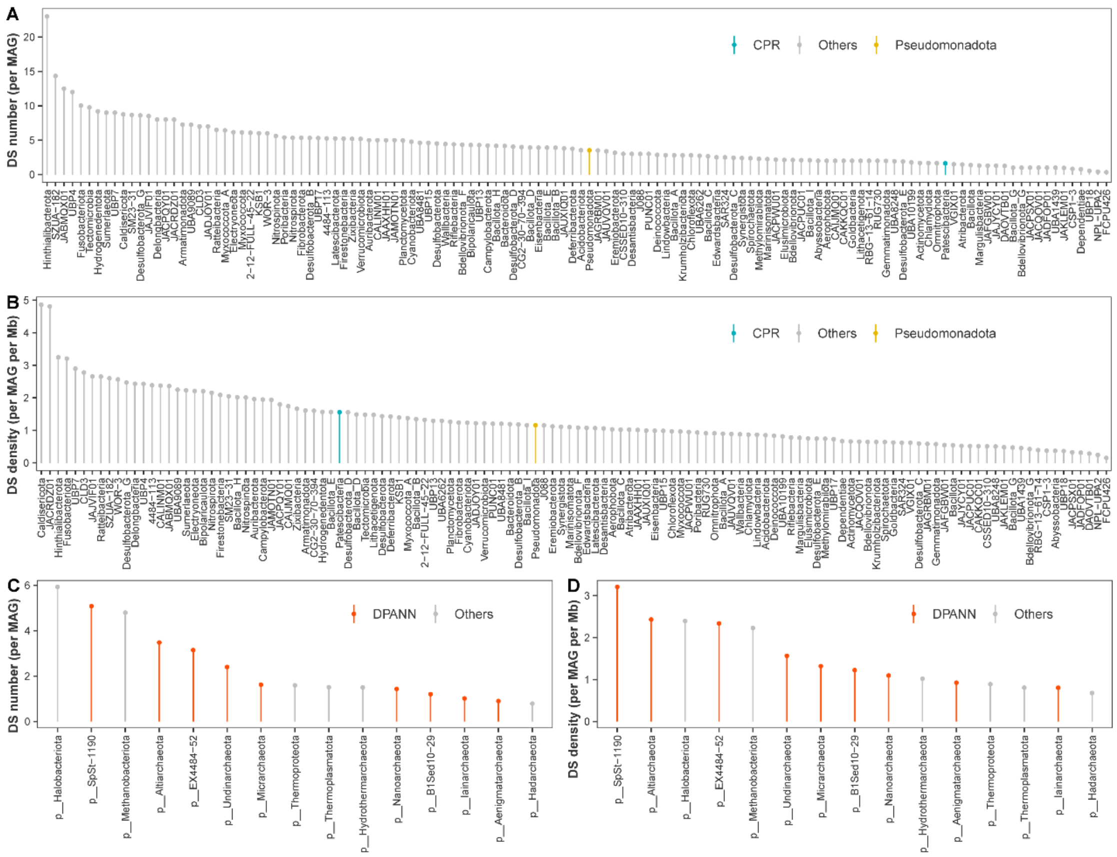

Fig. S10. Characteristics of defense systems (DSs) across microbial phyla. (A) Number of defense systems (per MAG) across bacterial phyla. **(B)** Density of defense systems (per MAG per Mb) across bacterial phyla. **(C)** Number of defense systems (per MAG) across archaeal phyla. **(D)** Density of defense systems (per MAG per Mb) across archaeal phyla. Source data are provided as a Source Data file.

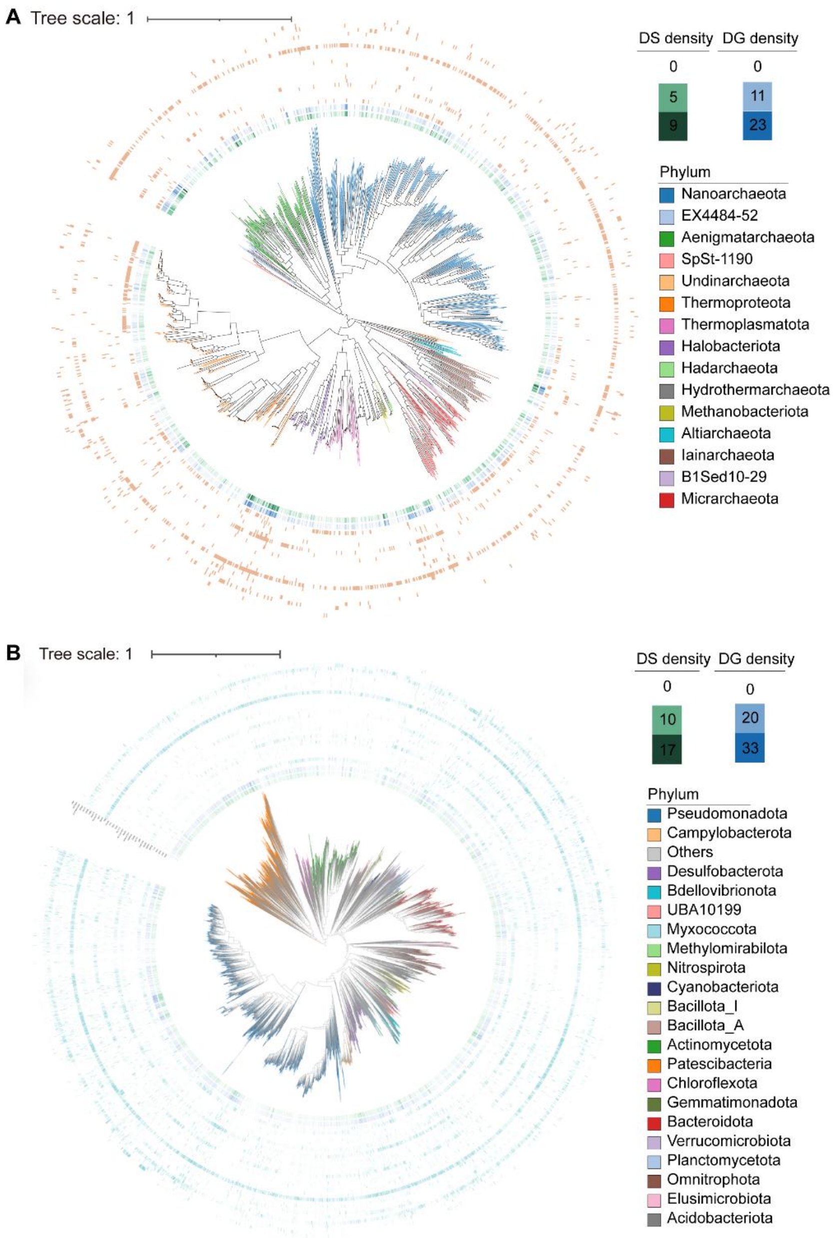

Fig. S11. Abundance and distribution of defense systems in groundwater prokaryotes. Phylogenetic trees of archaeal (A) and bacterial (B) genomes are categorized by their corresponding phyla. Defense systems density (DSs per Mb, green) and defense gene density (DGs per Mb, blue) are shown. The outer heatmap displays the presence or absence of defense families present in >0.5% of archaeal MAGs (A, 22 defense families) or >1% of bacterial MAGs (B, 33 defense families). In A (archaea), defense families from inner to outer rings are: AbiE, AbiU, BREX, CBASS, CapRel, CRISPR-Cas, DarTG, Gabija, Hachiman, Lamassu-Fam, MazEF, Mokosh, PD-T7-4, Paris, R-M, RIoC, SEFIR, Shedu, ShosTA, SoFIC, dCTPdeaminase, and pAgo. In B (bacteria), defense families from inner to outer rings are: AbiD, AbiE, AbiH, AbiU, Avs, BREX, CBASS, CapRel, CRISPR-Cas, DRT, DarTG, Gabija, Hachiman, Lamassu-Fam, MazEF, PD-Lambda-1, PD-T7-2, Paris, PfiAT, PrrC, Pycsar, R-M, Retron, RloC, RosmerTA, SanaTA, Septu, Shedu, SoFIC, Wadjet, dCTPdeaminase, dGTPase, and pAgo. Source data are provided as a Source Data file.

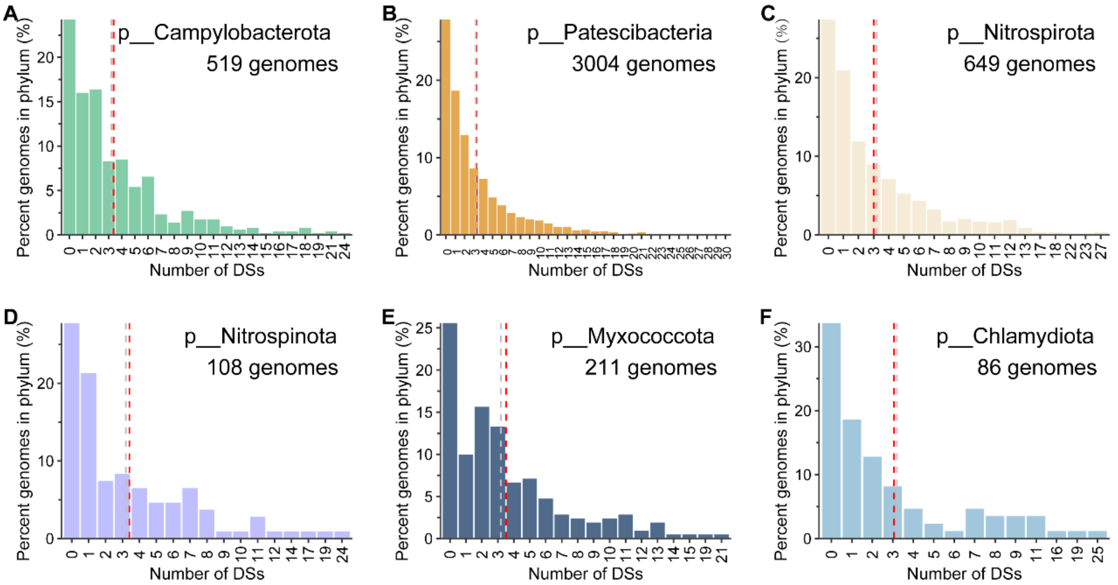

Fig. S12. Phylum-specific distribution of defense systems (DSs). The x-axis shows the number of defense systems per genome; the y-axis shows the fraction of genomes within each phylum. The dashed grey line indicates the average defense systems per genome (3.2) across 27,578 bacterial MAGs; while the dashed red line represents the average defense systems per genome within each phylum. Source data are provided as a Source Data file.

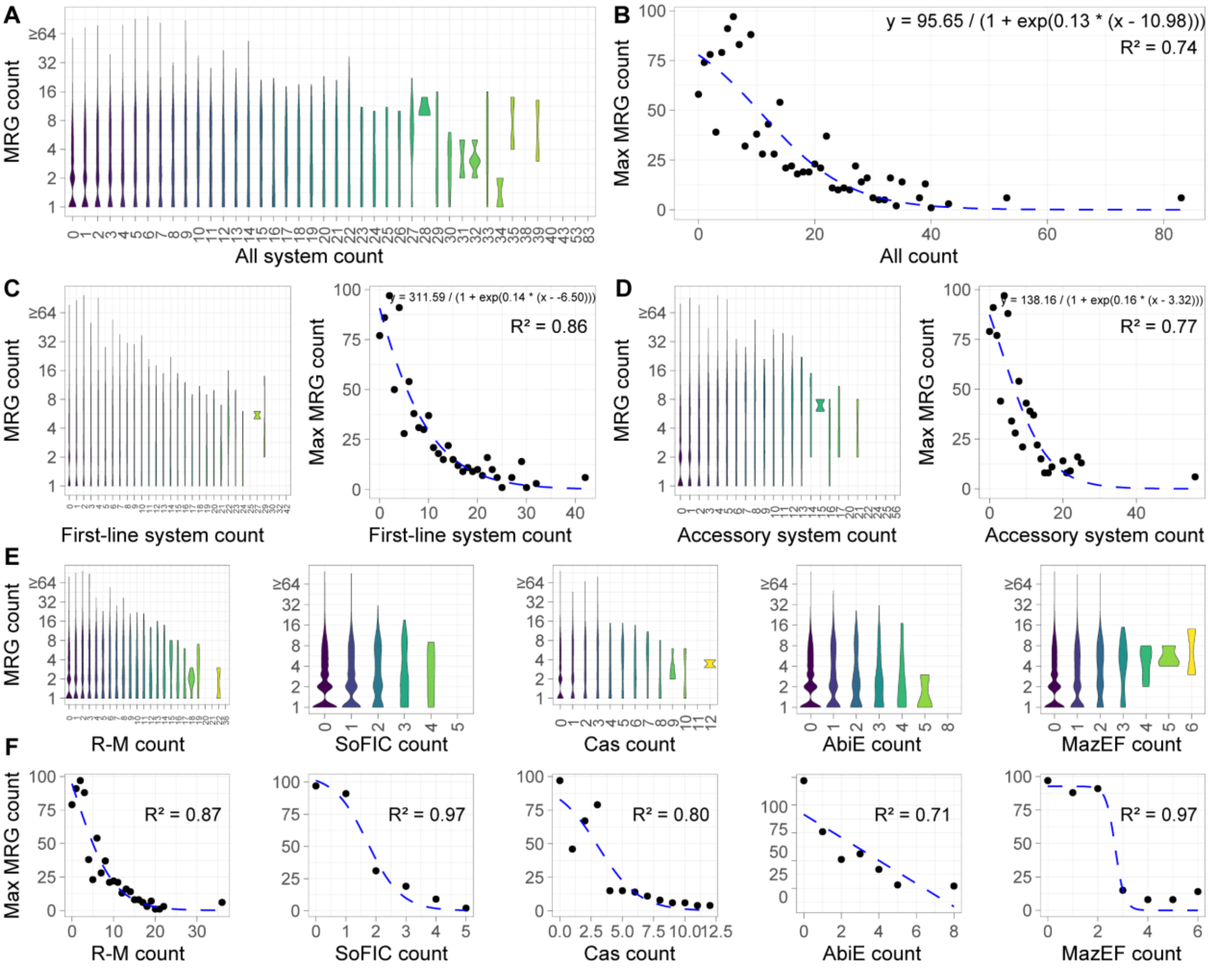

Fig. S13. Inverse relationship between defense systems and heavy-metal resistance genes (MRGs). (A) Change in MRG numbers with increasing defense system numbers across all bacterial genomes. **(B)** Decrease in the upper limit of MRG numbers as defense system numbers increases. **(C)** Relationship between MRG numbers and first-line defense system numbers. **(D)** Relationship between MRG numbers and accessory defense system numbers. **(E) (F)** Relationship between MRG numbers and the number of each first-line defense system (RM, SoFIC, CRISPR-Cas, AbiE, and MazEF). See Methods for detailed information of fitting models. Source data are provided as a Source Data file.

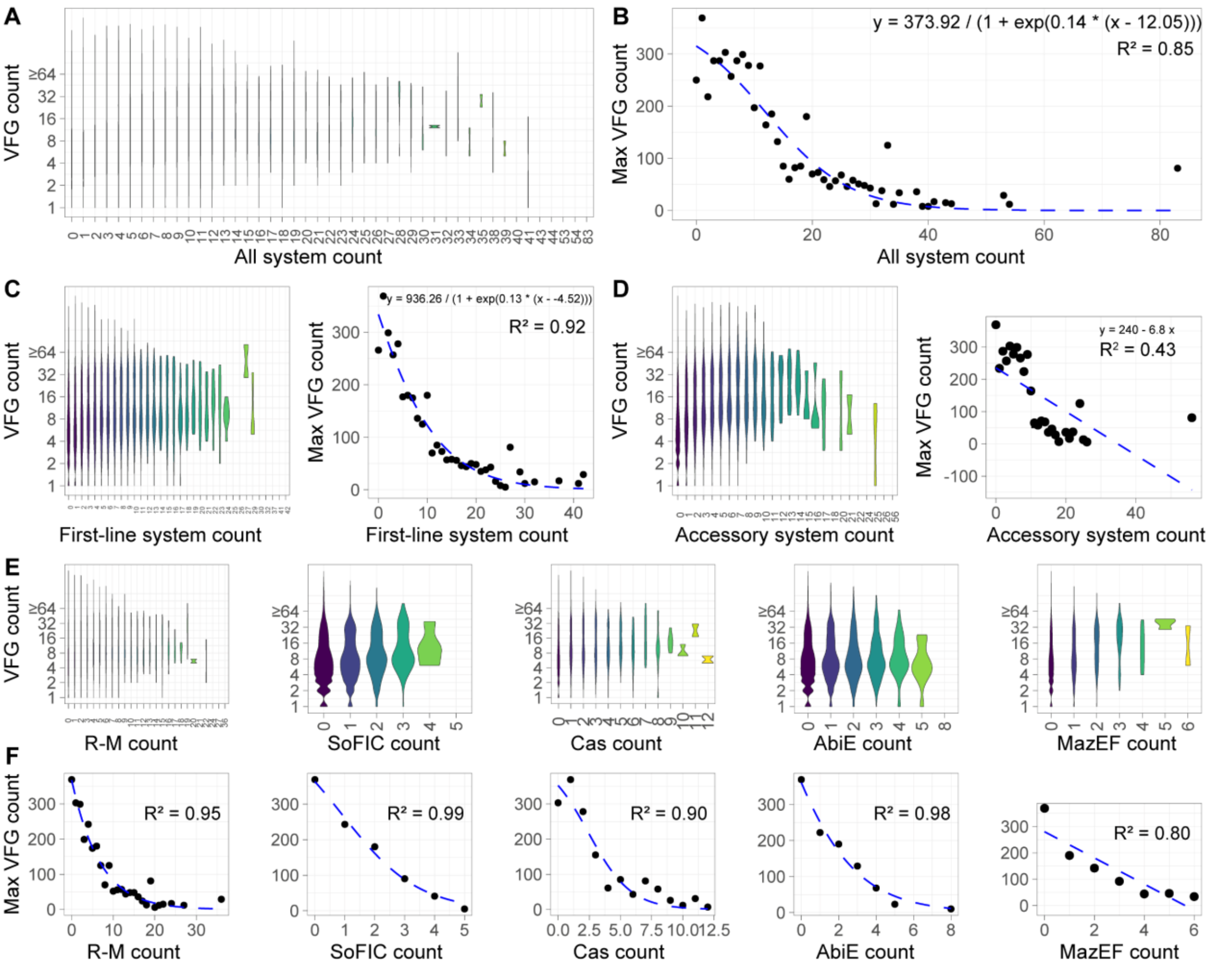

Fig. S14. Inverse relationship between defense systems and virulence factor genes (VFGs). (A) Change in VFG numbers with increasing defense system numbers across all bacterial genomes. **(B)** Decrease in the upper limit of VFG numbers as defense system numbers increase. **(C)** Relationship between VFG numbers and first-line defense systems numbers. **(D)** Relationship between VFG numbers and accessory defense system numbers. **(E) (F)** Relationship between VFG numbers and the number of each first-line defense system (RM, SoFIC, CRISPR-Cas, AbiE, and MazEF). See Methods for detailed information of fitting models. Source data are provided as a Source Data file.

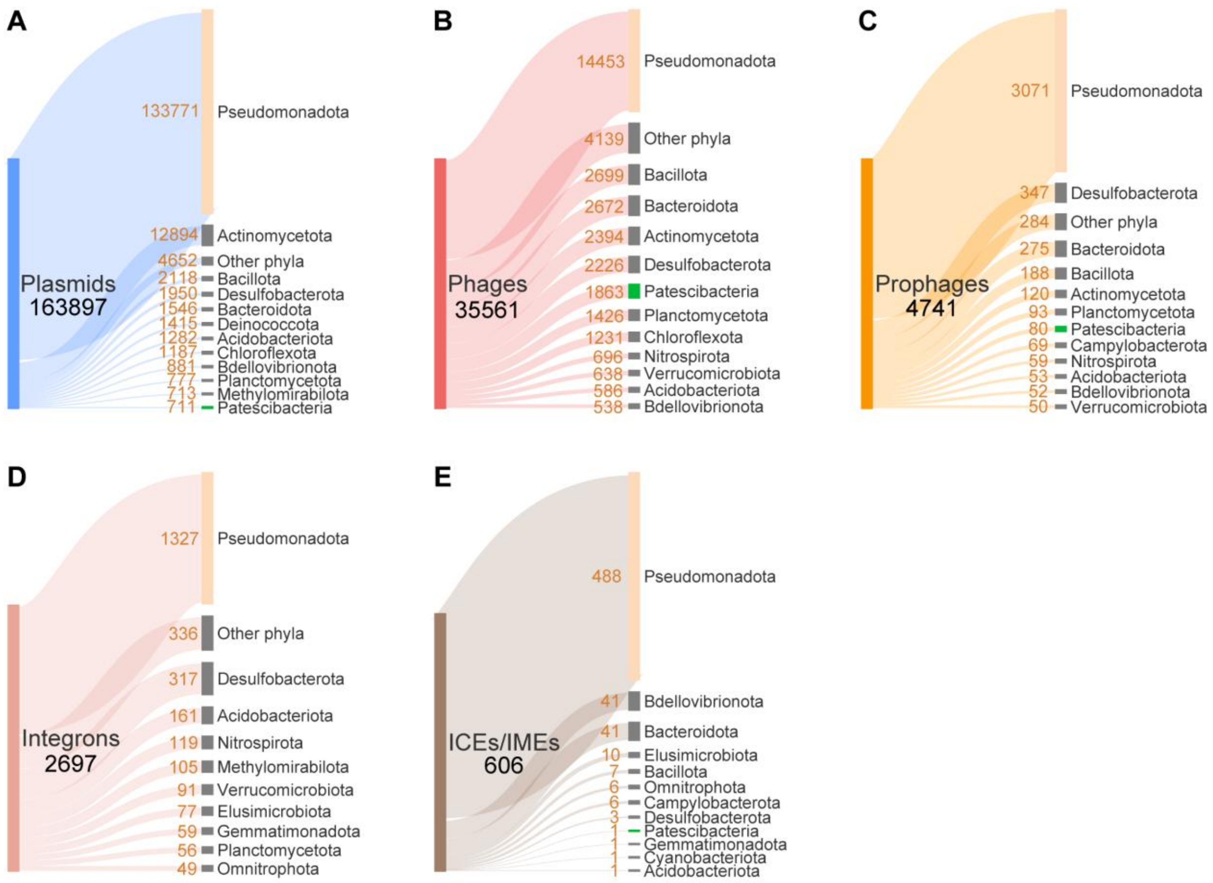

Fig. S15. Mobile genetic elements (MGEs) identified from bacterial genomes. For ease of visualization, phyla with fewer instances of specific MGEs were grouped into the category “Other phyla”. Source data are provided as a Source Data file.

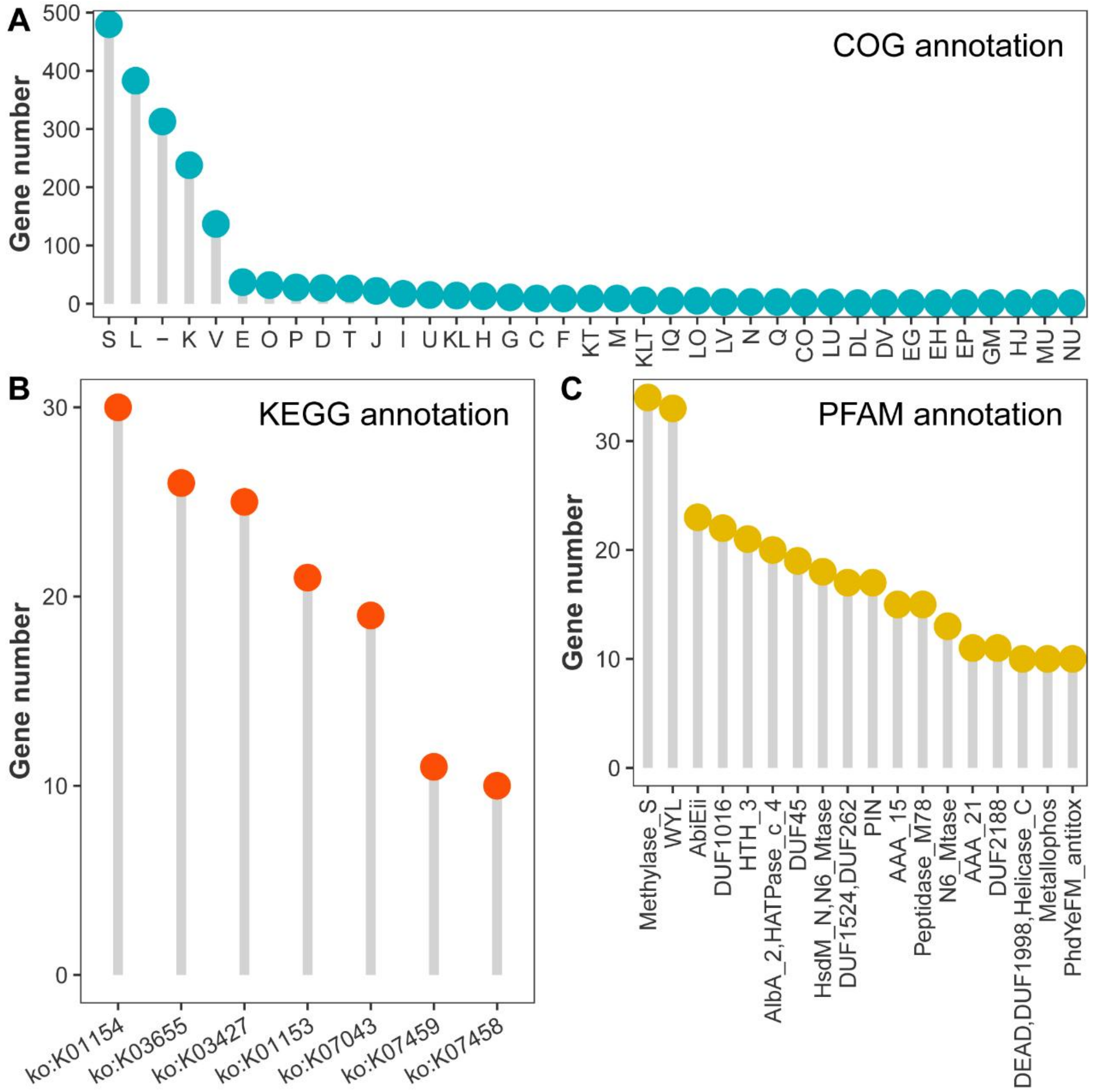

Fig. S16. Functional annotation of "non-defensive" genes in 662 bacterial defense islands. Genes were annotated using COG categories, KEGG, PFAM. Most genes lack KEGG and PFAM annotations (indicated by "–"), so this figure only shows the KEGG and PFAM categories with >10 annotated genes, excluding those marked with "–". Source data are provided as a Source Data file.

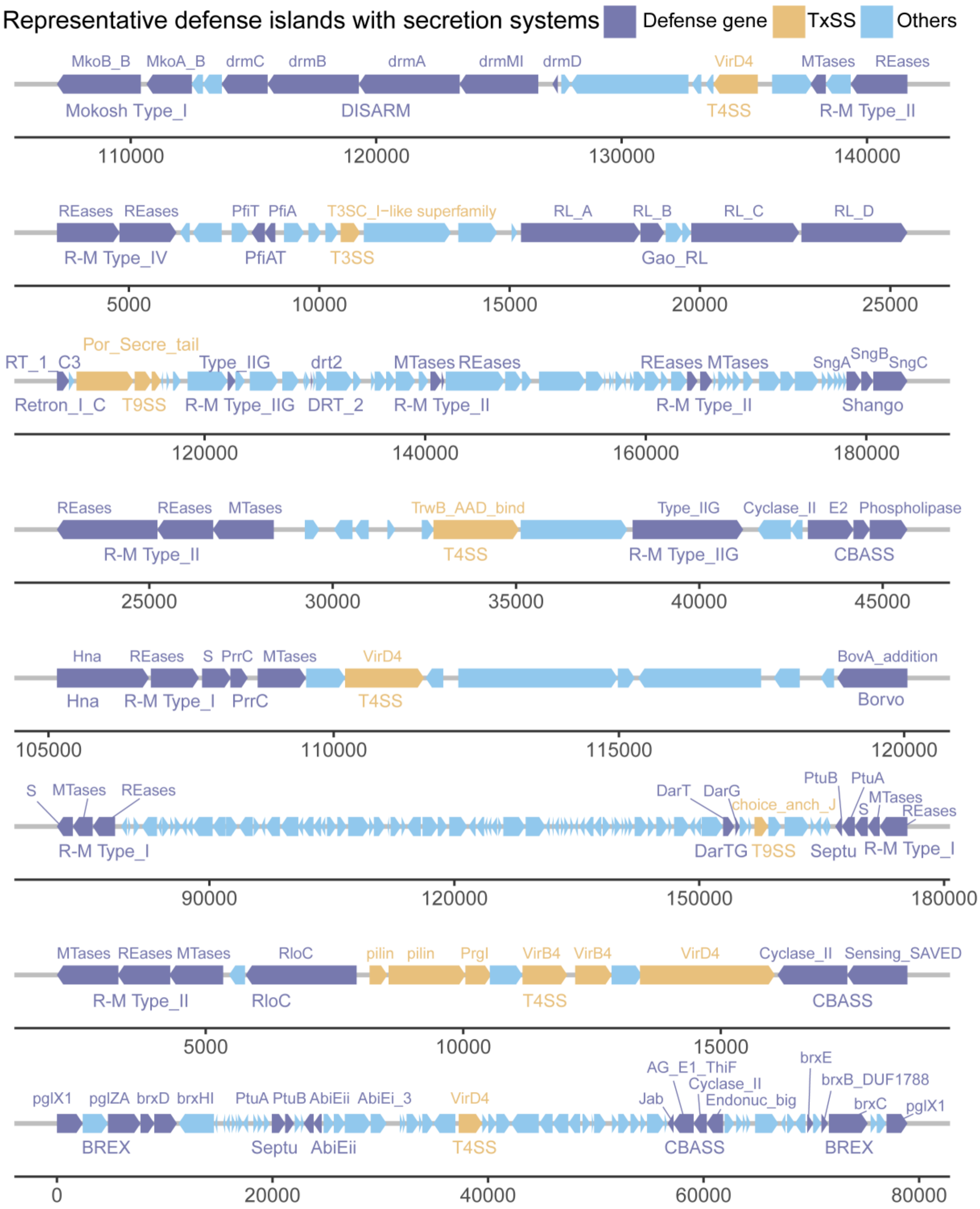

Fig. S17. Genomic contexts of defense genes and secretion systems in bacterial defense islands. Eight defense islands are shown, with distinct functional categories highlighted by colors. Source data are provided as a Source Data file.

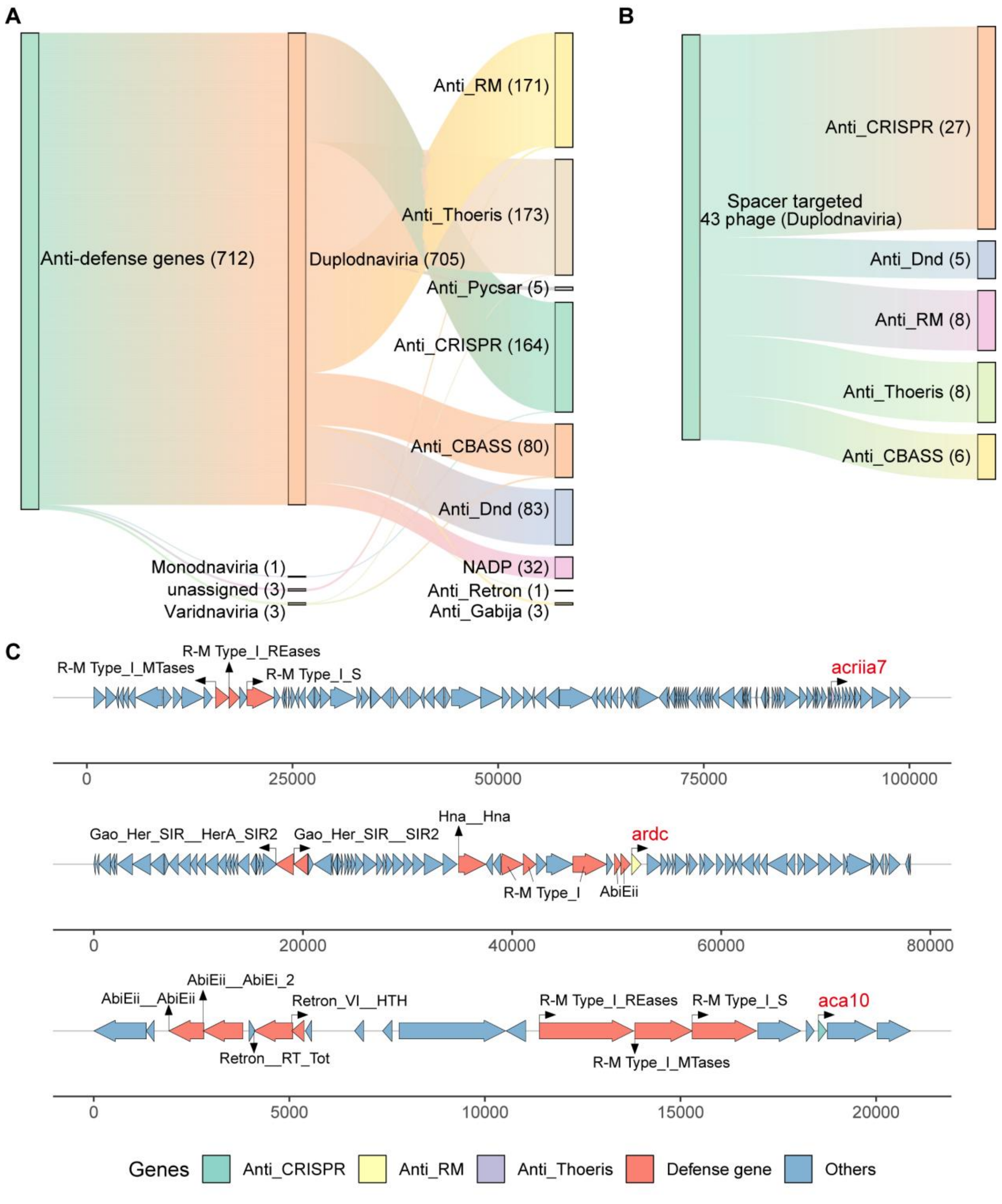

Fig. S18. Anti-defense genes of phages. **(A)** Distribution and types of anti-defense genes. **(B)** Composition of anti-defense genes in 43 phages targeted by bacterial spacers. **(C)** Examples of co-localization of defense and anti-defense genes in phages. Source data are provided as a Source Data file.

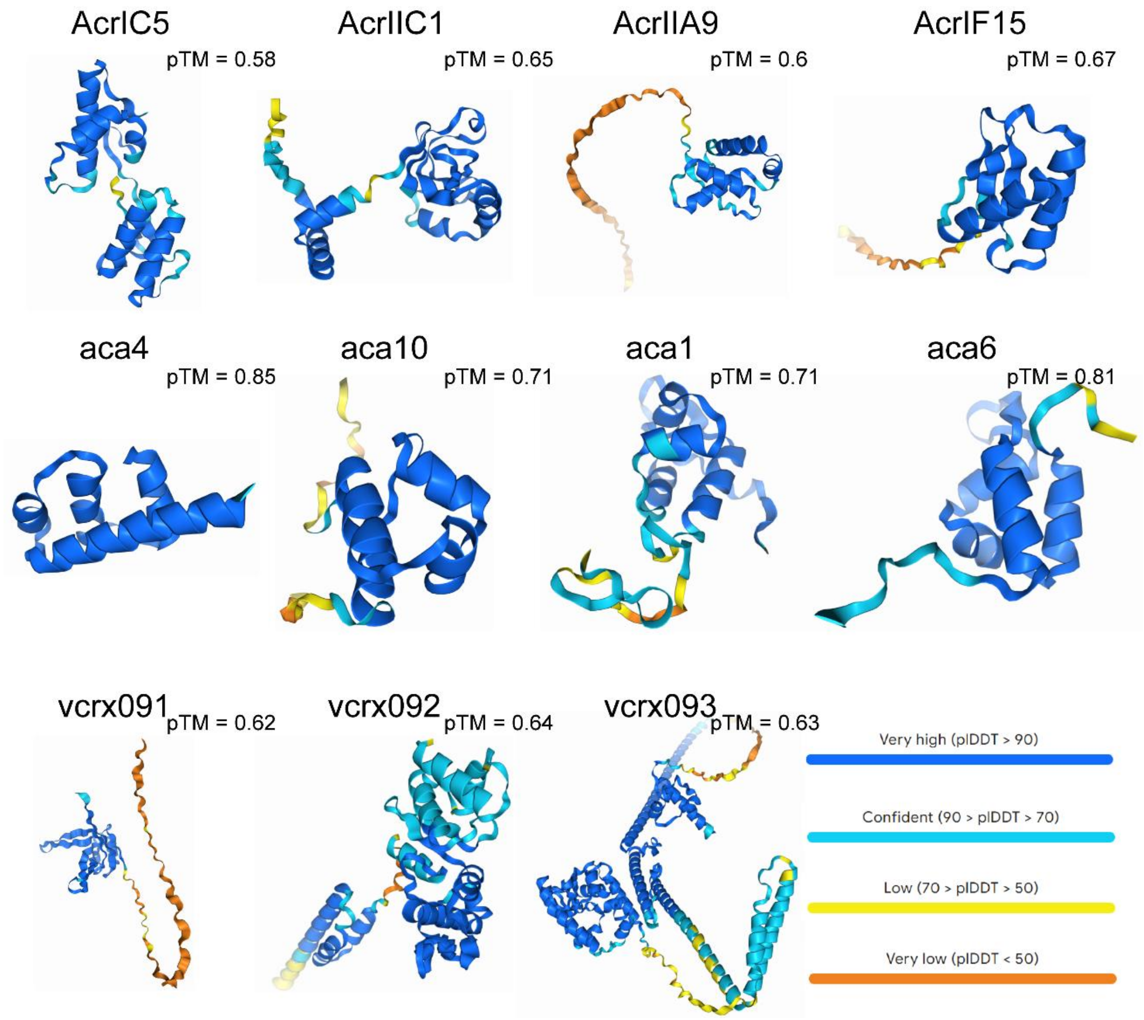

Fig. S19. Structure of representative phage anti-CRISPR proteins (targeted by host spacers). This includes anti-CRISPR (Acr) proteins, anti-CRISPR associated (aca) proteins, and host defence evasion (*hde*) operon (*vcrx091*-*vcrx093*) proteins. Their structures were predicted using AlphaFold3 with default parameters.

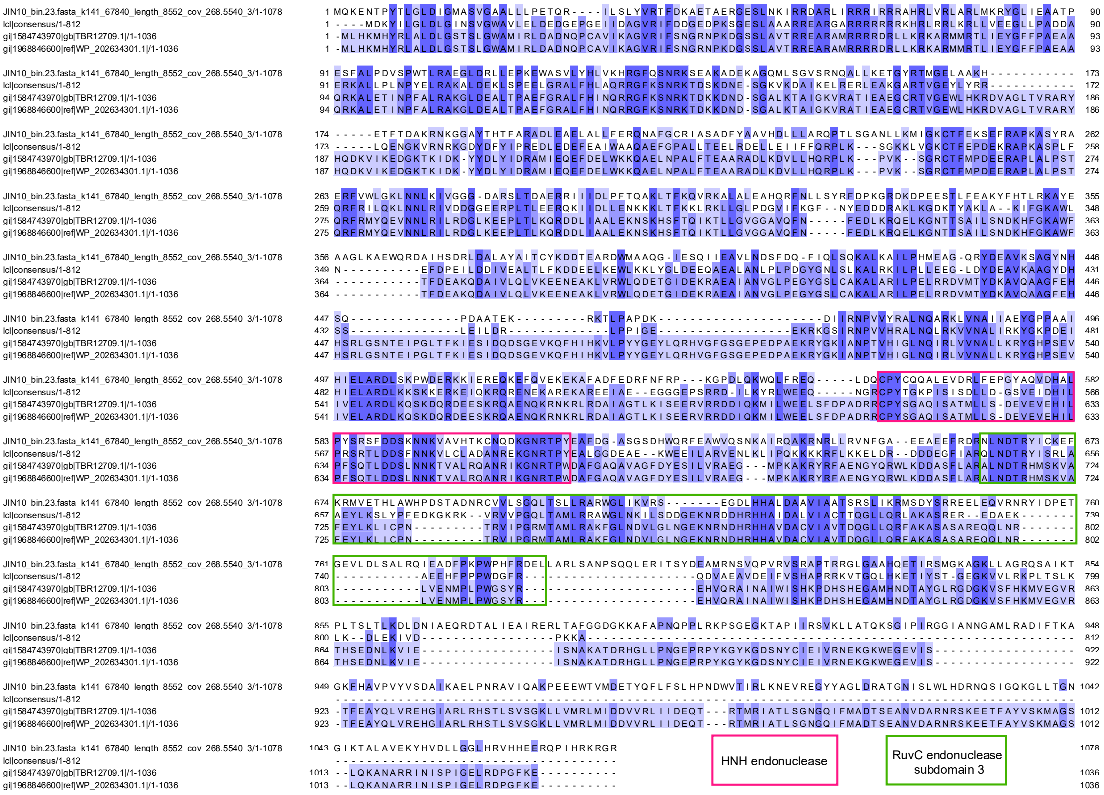

Fig. S20. Multiple sequence alignment of newly discovered Cas9, Cas9 from NCBI, and the COG3513 sequence from conserved domains database (CDD). The alignment was performed using Clustal Omega and visualized with Jalview. The sequences include: JIN10_bin.23.fasta_k141_67840_length_8552_cov_268.5540_3/1-1078, representing the Cas9 protein from *Rugosibacter sp.002422995* in groundwater; Ic||consensus/1-812, the COG3513 sequence from CDD; gi|1584743970|gb|TBR12709.1|/1-1036, the Cas9 from *Rugosibacter sp.*; and gi|1968846600|ref|WP_202634301.1|/1-1036, the Cas9 from *Rugosibacter aromaticivorans*.
