# Supplementary material for: The defensome of prokaryotes in aquifers
Source: Nat Commun. 2025 Jul 14;16:6482. doi: 10.1038/s41467-025-61467-w (PMC12259885; doi:10.1038/s41467-025-61467-w)
Supplement: Supplementary file 2 — Description of Additional Supplementary Files [file 41467_2025_61467_MOESM2_ESM.pdf]

Description of Additional Supplementary Files:

Supplementary Data 1: The detailed annotation and quality information of 27,578 bacterial MAGs.

Supplementary Data 2: Detailed annotation and quality information of 1,453 bacterial MAGs.

Supplementary Data 3: 172,333 defense systems from 22,818 NCBI RefSeq complete prokaryotic genomes.

Supplementary Data 4: The sensor, activator, and effector information of 152 defense systems.

Supplementary Data 5: Stepwise regression analysis of defense genes/systems and MAG size, MAG phylogenetic depth, and MAG N50.

Supplementary Data 6: MAG numbers of each phylum.

Supplementary Data 7: Virus-host ratios (VHR) of each phylum.

Supplementary Data 8: The fitting parameters of the logistic-like curve relating antibiotic resistance genes (ARGs), heavy-metal resistance genes (MRGs), and virulence factor genes (VFGs) to defense systems.

Supplementary Data 9: The detailed information about "first-line" defense systems and "accessory" defense systems.

Supplementary Data 10: Detailed information for experimental validated representative defense systems.
