# Supplementary material for: The defensome of prokaryotes in aquifers
Source: Nat Commun. 2025 Jul 14;16:6482. doi: 10.1038/s41467-025-61467-w (PMC12259885; doi:10.1038/s41467-025-61467-w)
Supplement: Supplementary file 4 — Reporting Summary [file 41467_2025_61467_MOESM4_ESM.pdf]

nature portfolio

Corresponding author(s): Jinren Ni

Last updated by author(s): Jun 14, 2025

Reporting Summary

Nature Portfolio wishes to improve the reproducibility of the work that we publish. This form provides structure for consistency and transparency in reporting. For further information on Nature Portfolio policies, see our Editorial Policies and the Editorial Policy Checklist.

Statistics

For all statistical analyses, confirm that the following items are present in the figure legend, table legend, main text, or Methods section.

n/a	Confirmed	
☐	☒	The exact sample size (*n*) for each experimental group/condition, given as a discrete number and unit of measurement
☐	☒	A statement on whether measurements were taken from distinct samples or whether the same sample was measured repeatedly
☐	☒	The statistical test(s) used AND whether they are one- or two-sided<br>*Only common tests should be described solely by name; describe more complex techniques in the Methods section.*
☒	☐	A description of all covariates tested
☐	☒	A description of any assumptions or corrections, such as tests of normality and adjustment for multiple comparisons
☐	☒	A full description of the statistical parameters including central tendency (e.g. means) or other basic estimates (e.g. regression coefficient) AND variation (e.g. standard deviation) or associated estimates of uncertainty (e.g. confidence intervals)
☐	☒	For null hypothesis testing, the test statistic (e.g. *F*, *t*, *r*) with confidence intervals, effect sizes, degrees of freedom and *P* value noted<br>*Give P values as exact values whenever suitable.*
☒	☐	For Bayesian analysis, information on the choice of priors and Markov chain Monte Carlo settings
☒	☐	For hierarchical and complex designs, identification of the appropriate level for tests and full reporting of outcomes
☐	☒	Estimates of effect sizes (e.g. Cohen's *d*, Pearson's *r*), indicating how they were calculated

Our web collection on statistics for biologists contains articles on many of the points above.

Software and code

Policy information about availability of computer code

Data collection	The Shell scripts and R codes used in this study have been made available in GitHub (https://github.com/lianmsu/aquifer-defensome) and Zenodo (https://doi.org/10.5281/zenodo.15656571)
Data analysis	metaWRAP v1.2.3; CheckM v1.1.2; dRep v2.5.4; OGT_prediction; GTDB-Tk v2.4.0; geNomad v1.8.0; IntegronFinder v2.0.5; ICEfinder v2.0; DefenseFinder v1.3.0; BLAST v2.9.0; Prodigal v2.6.3; diamond v2.0.6; DefensePredictor; eggNOG-mapper v2; NCBI CD-search; minced v0.4.2; AlphaFold3; ChimeraX; Clustal Omega; Muscle v5; trimAl; FastTree v2.1.11; Bowtie2; SAMtools; CoverM v0.3.1; R v4.3.1

For manuscripts utilizing custom algorithms or software that are central to the research but not yet described in published literature, software must be made available to editors and reviewers. We strongly encourage code deposition in a community repository (e.g. GitHub). See the Nature Portfolio guidelines for submitting code & software for further information.

Data

Policy information about availability of data

All manuscripts must include a data availability statement. This statement should provide the following information, where applicable:

- Accession codes, unique identifiers, or web links for publicly available datasets
- A description of any restrictions on data availability
- For clinical datasets or third party data, please ensure that the statement adheres to our policy

The metagenomic groundwater prokaryotic defensome catalogue (GPDC), viral anti-defense genes, phylogenetic trees of anti-CRISPR (Acr) genes based on amino acid sequences, Acr sequences, newly found CRISPR-Cas9 sequences, and protein structures are available in the Zenodo repository (https://doi.org/10.5281/

zenodo.13943877). All sequencing reads used in this study have been deposited in the NCBI database under accession code PRJNA858913. Source Data are provided with this paper.

Research involving human participants, their data, or biological material

Policy information about studies with human participants or human data. See also policy information about sex, gender (identity/presentation), and sexual orientation and race, ethnicity and racism.

Reporting on sex and gender	Not applicable.
Reporting on race, ethnicity, or other socially relevant groupings	Not applicable.
Population characteristics	Not applicable.
Recruitment	Not applicable.
Ethics oversight	Not applicable.

Note that full information on the approval of the study protocol must also be provided in the manuscript.

Field-specific reporting

Please select the one below that is the best fit for your research. If you are not sure, read the appropriate sections before making your selection.

☐ Life sciences ☐ Behavioural & social sciences ☒ Ecological, evolutionary & environmental sciences

For a reference copy of the document with all sections, see nature.com/documents/nr-reporting-summary-flat.pdf

Ecological, evolutionary & environmental sciences study design

All studies must disclose on these points even when the disclosure is negative.

Study description	Based on national campaigns of groundwater monitoring throughout China, microbial samples for metagenomic sequencing were collected to investigate the microbial ecology in the aquifer ecosystems. To this end, we collected 607 groundwater samples for metagenomic sequencing from 607 monitoring wells throughout China, and established the first Groundwater Prokaryotic Defense Catalogue (GPDC) to explore defensome in groundwater ecosystems. The same dataset have been mentioned and described in our previous work: Wu, Z. et al. Unveiling the unknown viral world in groundwater. Nat. Commun. 15, 6788 (2024).
Research sample	A total of 607 groundwater samples were collected from 525 newly constructed and 82 reconstructed monitoring wells throughout China between 2016 and 2017. The monitoring wells were distributed in seven distinct geological regions. The well depths varied between 0 m and 600 m, with diverse hydrological, geological contexts, and groundwater burial conditions. This represents a large-scale dataset of microbial metagenomes from groundwater.
Sampling strategy	The monitoring wells were distributed in seven geo-environmental zones (Northeast plain-mountain, Huanghuaihai and river delta Yangtze plain, South China bedrock low mountain foothill, Northwest loess plateau, Southwest China Karst rock mountain, Northwest arid desert, and Qinghai-Tibet plateau Alpine frozen soil) at depths ranging from of 0 to 600m. The layout of sampling sites was in accordance with Technical Specifications for Environmental Monitoring of Groundwater (HJ/T 164-2004), taking full consideration of hydrology, geological environment, and groundwater burial conditions. Therefore, the 607 groundwater samples collected in this study are representative, which could help to reflect groundwater prokaryotic defensome across China.
Data collection	Groundwater was pumped after well flushing and filtered through 0.22 m polycarbonate membranes (Millipore, USA) to capture microbial organisms. All filtered membranes were frozen at -80°C for high-throughput sequencing. Groundwater samples for physicochemical analysis were collected in 5 L sterile PET bottles and stored at -20°C. Total genomic DNA of 607 samples was extracted using the MoBio PowerSoil® kit (MoBio Laboratories, Carlsbad, CA, USA) following the manufacturer's protocol. DNA quantity and quality were determined using a NanoDrop Spectrophotometer (NanoDrop Technologies Inc., Wilmington, DE, USA). Genomic DNA wassequenced by Illumina HiSeq 4000 platform (Majorbio Company, Shanghai, China) with 2×150 bp paired-end reads. Generated metagenomic data are deposited in high performance computing platform of our research group. All Ni group members participated in the data collection process and the latitude, longitude, and elevation of each sampling site were recorded using a handheld GPS device.
Timing and spatial scale	All samples were collected from 607 groundwater monitoring wells throughout China (latitude: 18°-52°, longitude: 77°-133°) between 2016 and 2017. No gap in the collection periods. 607 monitoring wells are distributed in seven geo-environmental zones (Northeast plain-mountain, Huanghuaihai and river delta Yangtze plain, South China bedrock low mountain foothill, Northwest loess plateau, Southwest China Karst rock mountain, Northwest arid desert, and Qinghai-Tibet plateau Alpine frozen soil).
Data exclusions	All sequencing data were used for analyses without exclusion.
Reproducibility	The paper focuses on bioinformatics analyses, and all analyses can be reproduced using publicly available software packages provided in the Methods section. And the Shell scripts and R codes used in this study are available in GitHub.

Randomization	The study was designed to describe the microbial defensome from any possible condition. The work does not focus on the effect of environmental parameters.
Blinding	Blinding is not relevant to this study. This study is a massive descriptive discovery of previously unknown groundwater microbial defensome, blinding treatment does not impact the findings.

Did the study involve field work? ☒ Yes ☐ No

Field work, collection and transport

Field conditions	In this study, metagenomic sequencing was performed on 607 groundwater samples collected from 525 newly constructed and 82 reconstructed monitoring wells throughout China during 2016-2017. The monitoring wells were distributed in seven geo-environmental zones, including Northeast plain-mountain, Huanghuaihai and river delta Yangtze plain, South China bedrock low mountain foothill, Northwest loess plateau, Southwest China Karst rock mountain, Northwest arid desert, and Qinghai-Tibet plateau Alpine frozen soil.<br>The mean annual temperature of Northeast plain-mountain is 10-15°C, and the mean annual precipitation is between 500-1000 mm. The mean annual temperature of Huanghuaihai and river delta Yangtze plain is 13-18°C, and the mean annual precipitation is between 600-1500 mm. The mean annual temperature of South China bedrock low mountain foothill is 18-23°C, and the mean annual precipitation is between 1400-2000 mm. The mean annual temperature of Northwest loess plateau is 6-14°C, and the mean annual precipitation is between 300-600 mm. The mean annual temperature of Southwest China Karst rock mountain is 14-19°C, and the mean annual precipitation is between 1000-2000 mm. The mean annual temperature of Northwest arid desert is 8-14°C, and the mean annual precipitation is between 50-200 mm. The mean annual temperature of Qinghai-Tibet plateau Alpine frozen soil is -5-5° C, and the mean annual precipitation is between 500-1000 mm.
Location	Groundwater samples were collected from monitoring wells across China (latitude: 18°-52°, longitude: 77°-133°) at depths ranging from 0 to to 600 m.
Access & import/export	All groundwater sampling followed China's standard procedure for the environmental monitoring of groundwater (HJ494-2009). Groundwater monitoring wells were constructed by China Institute of Geo-environmental Monitoring (CIGEM). We got permission from CIGEM to enter these areas for sampling.
Disturbance	No disturbances were caused by this study.

Reporting for specific materials, systems and methods

We require information from authors about some types of materials, experimental systems and methods used in many studies. Here, indicate whether each material, system or method listed is relevant to your study. If you are not sure if a list item applies to your research, read the appropriate section before selecting a response.

Materials & experimental systems

n/a	Involved in the study
☒	☐ Antibodies
☒	☐ Eukaryotic cell lines
☒	☐ Palaeontology and archaeology
☒	☐ Animals and other organisms
☒	☐ Clinical data
☒	☐ Dual use research of concern
☒	☐ Plants

Methods

n/a	Involved in the study
☒	☐ ChIP-seq
☒	☐ Flow cytometry
☒	☐ MRI-based neuroimaging

Plants

Seed stocks	Not applicable.
Novel plant genotypes	Not applicable.
Authentication	Not applicable.
